# Quality of Life Perception among Portuguese Celiac Patients: A Cross-Sectional Study Using the Celiac Disease Questionnaire (CDQ)

**DOI:** 10.3390/nu15092051

**Published:** 2023-04-24

**Authors:** Cláudia Chaves, António Raposo, Renata Puppin Zandonadi, Eduardo Yoshio Nakano, Fernando Ramos, Edite Teixeira-Lemos

**Affiliations:** 1 ESSV, Centre for Studies in Education and Innovation (CI&DEI), Polytechnic University of Viseu, 3504-510 Viseu, Portugal; claudiachaves21@gmail.com; 2 CBIOS (Research Center for Biosciences and Health Technologies), Universidade Lusófona de Humanidades e Tecnologias, Campo Grande 376, 1749-024 Lisboa, Portugal; 3 University of Brasília, Faculty of Health Sciences, Nutrition Department, Campus Universitário Darcy Ribeiro, Brasília 70910-900, Brazil; 4 University of Brasília, Department of Statistics, Brasília 70910-900, Brazil; eynakano@gmail.com; 5 Faculty of Pharmacy, University of Coimbra, Azinhaga de Santa Comba, 3000-548 Coimbra, Portugal; framos@ff.uc.pt; 6 Associated Laboratory for Green Chemistry (LAQV) of the Network of Chemistry and Technology (REQUIMTE), Rua D. Manuel II, Apartado 55142, 4051-401 Porto, Portugal; 7 CERNAS-IPV, Research Centre, Polytechnic University of Viseu, 3504-510 Viseu, Portugal

**Keywords:** celiac disease quality of life questionnaire, quality of life, celiac patient, gluten-free diet

## Abstract

The aim of this study is to assess Portuguese celiac patients’ quality of life (QoL) perception. A cross-sectional study was performed with a non-probability convenience sample of Portuguese celiac patients using an online self-administered celiac disease quality of life questionnaire (CDQ), previously validated for the Portuguese population. The final sample comprised 234 celiac patients, which included the following: primarily women (69.2%); aged between 18 and 49 years old (56.4%); with a partner (60.6%); with a high educational level (58.9%—graduated or post-graduated); following a gluten-free diet (GFD) (55.1%); and not using antidepressants (93.1%). The Portuguese CDQ presented good reliability and responsiveness in this sample of Portuguese celiac patients. In general, the CDQ in Portugal was affected by age at diagnosis (*p* = 0.017), educational level (*p* = 0.005), and compliance with GFD (*p* = 0.034). The emotion domain was affected only by using antidepressants (*p* = 0.036). The social domain was affected by gender (females had lower rates, *p* = 0.016), age at diagnosis (*p* = 0.009), educational level (*p* = 0.000), and compliance with a GFD (*p* = 0.002). The worries domain did not differ according to socioeconomic data. The symptoms domain was affected by compliance with GFD (*p* = 0.000), age at diagnosis (*p* = 0.000), and educational level (*p* = 0.014). Data on celiac QoL is essential to support the formulation and implementation of strategies to minimize the issues suffered by celiac patients, lowering their physical, emotional, and social burden. Additionally, data on Portuguese celiac disease patients using the CDQ will allow future comparative research among celiac populations from different countries.

## 1. Introduction

As has been well established in the literature, celiac disease (CD) is an immune-mediated enteropathy associated with gluten consumption in genetically predisposed individuals. Considered a public health problem worldwide, affecting approximately 1% of the world population [1], the prevalence of CD has been increasing worldwide over the last decades, and Portugal is no exception [2,3]. Manifestations of the disease might include diarrhoea, abdominal pain and distension, constipation, flatulence, weight loss, fatigue, depression, anaemia, epilepsy, ataxia, osteopenia, and osteoporosis, among others that affect health and quality of life [4,5,6]. Although CD commonly combines many symptoms, some individuals do not present symptoms, even in cases of mucosal damage [7]. Those asymptomatic individuals have a high risk of complications since they do not recognize the clinical aggravation of CD and tend to present more resistance to treatment [7].

CD treatment consists of adherence to a lifelong gluten-free diet (GFD) characterized by excluding cereal grains (wheat, rye, barley, and, in some cases, oats) and their derivatives from the diet [8]. Rigorous adherence to the diet leads to the remission of symptoms, damage to the intestinal mucosa, and serological normalization, reducing the risk of cancer and other previously mentioned conditions resulting from untreated CD [9,10]. Left untreated, CD can lead to severe health problems and increased mortality. Moreover, it is known that chronic disorders impact patients’ QoL [7,11,12,13]. Since the treatment of CD is essentially dietary, an individual with CD faces several daily difficulties in addition to removing gluten from their diet. From social issues (as they eat differently from the people they live with) to difficulties in accessing gluten-free foods, as well as several other obstacles to adherence to dietary treatment. Therefore, dietary restrictions, social exclusion, and the symptoms of the disease can significantly influence the commitment to treatment, the way the patients relate to food, and, consequently, their quality of life [7,11,12,13,14].

Quality of life has been in the spotlight in clinical practice and research into promoting more effective treatments and public policies for individuals and populations. According to the World Health Organization (WHO), quality of life (QoL) is defined as “an individual’s perception of their position in life in the context of the culture and value systems in which they live and in relation to their goals, expectations, standards, and concerns” [15] and health is defined as “a state of complete physical, mental, and social well-being” [16]. Therefore, according to the WHO, in order to achieve the aim of striving towards optimal health, it is essential to understand the patient’s perception of quality of life [17]. For this reason, the concept of health-related quality of life (HRQOL) has emerged. This is “an individual’s or a group’s perceived physical and mental health over time” [18] and includes the impact of health or disease on the individual’s ability to live a fulfilling life. Considering that quality of life is a multidimensional concept that comprises subjective evaluations of positive and negative aspects of life regarding individuals’ health, objectives, expectations, standards, and concerns [19], understanding QoL perception is important to evaluate and implement policies that can reduce the physical, emotional, and social burden on the individual affected by a disease [20,21].

In recent years, concern about celiac patients’ QoL has increased [22,23,24,25,26,27] and to measure the impact of the difficulties faced by individuals with CD on their QoL, an instrument was developed and validated, considering the specificities linked to the experience and management of CD [8]. The Celiac Disease Quality of Life Questionnaire (CDQ) is a quantitative, self-administered questionnaire comprising 28 questions to evaluate QoL in CD individuals that consider symptoms, diet, social exclusion, and concerns experienced by celiac patients [11]. The CDQ is an essential and cost-effective questionnaire to understand aspects related to the QoL of celiacs and their daily choices, mental health and well-being, and the social limitations imposed by this chronic disease because of their lifelong changes in lifestyle and diet [20,28].

CDQ was first applied in Germany [11] and later translated and applied in other countries, such as Italy [6], Spain [29], France [30], Turkey [28], the United States [31], Brazil [12], India [32], Poland [8], Iran [33], Australia [34], Argentina [35], Morocco [36], Hungary [37], among others. In Portugal, a study translated the CDQ into Portuguese and validated it [38], but no recent national data on CD quality of life exists. Therefore, this is the first study on Portuguese CD individuals’ QoL using the same questionnaire used in other countries, which is essential for comparisons among different populations regarding a specific health problem such as CD. Therefore, the aim of this study is to assess Portuguese celiac patients’ QoL perceptions using the CDQ. Considering that Portugal is one of the countries that has more public policies for celiac patient support [39], we hypothesize that celiacs from Portugal present high CDQ scores for QoL perception. We expect our results will allow later comparative research among celiac populations from different countries and assist health professionals and the government in promoting practical strategies to improve Portuguese celiac patients’ QoL.

## 2. Materials and Methods

### 2.1. Study Design, Sampling, and Instruments

This cross-sectional study was conducted using non-probabilistic convenience sampling, in which people with celiac disease completed the online questionnaire in 2022. This method was selected considering the COVID-19 pandemic during the data collection, limiting the possibility of a face-to-face survey. Studies have shown that it is an effective, efficient, and low-cost way to recruit study participants and allows for a larger sample size and a shorter completion time [40,41]. A self-administered instrument to evaluate the CD patients’ quality of life (CDQ), developed by Häuser et al. [11] and validated for the Portuguese population by Lobão et al. [38] was used. The CDQ consists of 4 domains with 7 items each (emotions, gastrointestinal symptoms, concerns, and social), totalling 28 items. The items were evaluated using a 7-point scale (from “1”—worst QoL perception to “7”—best QoL perception), in which the best possible final score is 196 points (reflecting the best QoL perception level). Sociodemographic characteristics such as gender, age, marital status, educational level, and clinical variables such as age at diagnosis of CD, compliance with the gluten-free diet, and use of antidepressants were also collected.

### 2.2. Participants and Ethics

An online questionnaire was completed through the SurveyMonkey^®^ ( Momentive AI, San Mateo, CA, USA) online platform, from February to May 2022. Participants were recruited nationwide by invitation by the *Associação Portuguesa de Celíacos* (APC) or via social networks where the access link was posted. The following inclusion criteria were considered: (a) individuals with a self-reported correct diagnosis for CD (according to the European Society Paediatric Gastroenterology, Hepatology, and Nutrition criteria [42]), (b) adults (over 18 years old), and (c) celiac patients residing in Portugal. Celiac patients who agreed to participate in the study were directed to the questionnaire items. Patients who did not want to participate were directed to the end of the page and thanked for their time. A total of 234 celiac patients agreed to participate in the study and completed the questionnaire.

This study followed the APA Ethical Guidelines for Research with Human Subjects; all participants were fully informed about the general scope of the study, informed consent was obtained from the participants, and no compensation was provided for participation. The Polytechnic Institute of Viseu ethics committee granted ethical clearance for this study (n.º 59/SUB/2021).

### 2.3. Statistical Analysis

Data extracted from the SurveyMonkey^®^ platform were analyzed by IBM SPSS Statistics for Windows, version 22 (IBM Corp., Armonk, NY, USA). The statistical analysis considered the CDQ scores, with higher scores indicating a higher quality of life. Missing values in the dimensions were replaced with the median value. If more than 20% of the questions were left blank, the individual was excluded from the analysis. The total CDQ score was determined for each individual along with their sociodemographic characteristics.

Descriptive statistics such as the mean, median, standard deviation, floor, and ceiling effects of the CDQ domains were calculated. Student’s *t*-test and Analysis of Variance (ANOVA) followed by Tukey’s posthoc test were used to compare the CDQ domains with the variables of interest. All tests considered two-tailed hypotheses with a significance level of 5%. The internal consistency of the CDQ domains was assessed using Cronbach’s alpha.

## 3. Results

### 3.1. Sample Characteristics

The final sample comprised 234 celiac patients, primarily women (69.2%); aged between 18 and 49 y/o (56.4%); with a partner (60.6%); with a high educational level (58.9%—graduated or post-graduated); following a GFD (55.1%); and not using antidepressants (93.1%).

### 3.2. Reliability Analysis of the Instrument

The internal consistency of the CDQ and its domains were evaluated through Cronbach’s alpha (Table 1). All the CDQ domains had good reliability (α > 0.7), as well as the complete instrument. The instrument as a whole presents good responsiveness (floor and ceiling effects < 5%), indicating it is sensitive to detect differences in the division of responsibility between participants located at the extremes (e.g., with better or worse scores) [43].

### 3.3. Participants’ Quality of Life by Sociodemographic Data—CDQ

CDQ data were evaluated using sociodemographic data (Table 2). In general, CDQ was affected by age at diagnosis (*p* = 0.017), educational level (*p* = 0.005), and compliance with GFD (*p* = 0.034). The emotion domain was affected only by using antidepressants (0.036). The social domain was affected by gender (females had lower rates, *p* = 0.016), age at diagnosis (*p* = 0.009), educational level (*p* = 0.000), and compliance with a gluten-free diet (*p* = 0.002). The worries domain did not differ based on socioeconomic data. The symptoms domain was affected by compliance with GFD (*p* = 0.000), age at diagnosis (*p* = 0.000), and educational level (*p* = 0.014).

## 4. Discussion

This is a recent study performed on the evaluation of QoL in Portuguese celiac patients using the CDQ validated for the Portuguese population [38]. The perception of QoL in celiacs has garnered significant interest among health professionals and researchers generating data to guide public policies and treatments [11,12,21,28,29,44], since the burden of symptoms and a lifelong GFD are significant factors in celiacs’ lives. A study showed these burdens are more highly perceived than in many other chronic illnesses [45]. It reinforces the importance of using a disease-specific questionnaire when evaluating the QoL of celiac patients.

This study of Portuguese CD individuals’ QoL was performed during the COVID-19 pandemic. A previous survey validating CQD in Portuguese was conducted in 2011 in Portugal [38] and presented a similar number of participants (*n* = 231) to our study (*n* = 234). The authors found a similar Cronbach’s alpha (0.910) [38] to ours (0.956) for CDQ. However, the study did not show the results of QoL obtained by the CDQ or sociodemographic characteristics, which prevented us from making direct comparisons. Our final sample comprised primarily females (*n* = 162; 69.2%), similar to previous studies conducted to evaluate CD patients’ QoL [12,20,28,35,46,47], which, in part, can be justified by the higher incidence of CD in females than in males [48]. Contrary to our hypothesis, Portuguese celiac patients did not present high CDQ scores. The mean of the total CDQ score obtained was 103.28, far from the maximum score (197) and lower than mean scores found in studies performed in other countries, such as Germany (mean score = 151) [20], Italy (mean score = 159) [6], Australia (mean score = 147) [34], Turkey (mean score = 124) [28], Argentina (mean score = 124) [35], Iran (mean score = 119) [33], and Brazil (mean score = 119) [12]. Moreover, the sub-optimal scores, especially in the social domain, are concerning. One possible explanation could be the period of data collection that occurred during the COVID-19 pandemic, but we do not have previous data to compare to the period before the pandemic in Portugal. Theoretically, staying at home favours the time to eat and prepare meals, potentially favouring following a strict gluten-free diet [49,50]. This could potentially reduce the fear of consuming products that contain gluten, but negatively impact the social domain (as occurred in our study) due to the isolation claim during the COVID-19 pandemic. A study confirmed that most CD patients felt it was easier to follow the GFD during the pandemic isolation period, consuming meals at home, indicating that eating out is perceived by celiacs as a risk for gluten contamination [50]. However, further study is necessary to verify how the COVID-19 pandemic affected Portuguese celiac patients and compare the periods before, during, and after the pandemic.

Gender only influenced QoL perception in the CDQ social domain, in which males presented higher scores than females (Table 1), probably due to the greater concern of females with health and diet [12,51,52,53,54]. It possibly makes them perceive greater social restrictions when they have CD and follow a GFD. Similar results were found in a study performed on 674 Brazilian celiac patients during the COVID-19 pandemic, but the authors did not find differences among genders in the social domain or in the other CDQ domains [55]. In contrast to our findings, studies showed that CD females present lower levels of QoL than males [56,57,58,59], higher distress due to daily life restrictions, and a higher burden with CD than males [57]. Furthermore, a previous study with 195 Portuguese celiac patients evaluating health-related quality of life using the SF-36 questionnaire [60] showed that males presented higher scores for health-related quality of life than females for all SF-36 domains, not only the social domain. However, it is important to mention that SF-36 is a generic health-related QoL [61,62], not specific to CD patients, which may justify the difference between the results. Due to the specificity of CD, it is essential to use specific instruments to measure the QoL of individuals with CD, which contemplate aspects of the clinical manifestations of CD and the difficulties faced with GFD [20,63]. Therefore, the use of different questionnaires (generic and specific) to measure health-related quality of life (HRQoL) may impact the results, as found when comparing the studies conducted in Portugal with different instruments. Therefore, CD population-specific validated questionnaires are considered the most reliable for evaluating CD patients’ QoL since they include their struggles and disease specificities [20,63]. Gender, age, and marital status not influencing CD patients’ general CDQ scores were not expected because previous studies with non-celiac individuals have shown that younger and female individuals were at higher risk for distress and those living with a partner had better QoL than those with no partner [64,65,66,67].

Similar to other studies [44,58,68,69], the longer time since the diagnosis was related to higher scores on the social domain and total CDQ. In our study, patients with a CD diagnosis up to 20 y/o presented the best score not only for total CDQ and social domain but also for the symptoms domain. It probably occurred because these individuals live longer with CD and cope better with celiac disease, its treatments, and its symptoms, but this study did not evaluate it. A study [68] showed that CD patients had a lower QoL at the time of diagnosis than those with a longer diagnosis period. Another study [69] with patients newly diagnosed compared with those with a longer diagnosis showed that patients with a longer diagnosis had better QoL, indicating that there is a better adaptation to the restrictions imposed by the treatment [69].

Studies have demonstrated that higher educational levels contribute to the patient’s physical and social function, health perception, and mental health, reducing the adverse effects of many chronic medical conditions [70,71,72,73]. Our results did not corroborate the above findings, in which individuals with lower educational levels presented better CDQ scores than those with the highest educational levels. It is evident that our sample was mainly composed of celiacs with high education levels, which can be considered a potential limitation of our study. Further research should be performed to evaluate the QoL of CD individuals with lower education levels and their potential influence on their QoL.

Notably, 71.4% (*n* = 25) of the participants with an educational level up to primary school and 55.7% (*n* = 34) with a secondary school education do not always follow the GFD. In comparison, almost 70% of post-graduate participants consistently follow the GFD. Compliance with a GFD can potentially be the reason for the difference in QoL perception [74]. Although compliance with a GFD positively impacts the remission of symptoms and reduces the risk of complications derived from untreated CD, it might cause more concerns about eating (fear of gluten contamination, difficulty finding gluten-free products, high price of products) and having more difficulty with social interaction, mainly related to events involving food, influencing QoL perception [39,75,76,77].

Almost half of our participants do not always follow a GFD (44.9%). This result was not expected since a GFD is currently the only effective treatment to control CD and its repercussions on celiac patients’ health [75,77], but it is similar to what was found in a study performed in Argentina [35], in which only 53% of the sample followed a strict GFD, and in Turkey, in which only 60% of participants reported following the GFD, and those on a GFD presented higher CDQ scores than those who were not on a GFD [28]. A previous study performed in Portugal [60] showed that approximately 97% of participants reported trying to comply with the GFD, but almost 48% sometimes ingested gluten, in line with our findings. Stratifying our sample, CD patients who never follow a GFD are those with the worst results for the symptoms domain, and those who follow the GFD irregularly have the best results (*p* = 0.001). This surprising result differs from other studies in which celiacs on a strict GFD presented higher QoL in the symptoms domain than those who did not follow a strict GFD. This is likely because celiacs not complying with a strict GFD suffer from many gastrointestinal manifestations, resulting in reduced QoL [78,79,80]. It is worth mentioning that celiac patients not adhering to a strict GFD presented the best CDQ scores in the total, social, and symptoms domain (Table 2). Despite not being evaluated in this study, celiac patients not following a GFD are probably asymptomatic, which might influence CDQ scores for the symptoms’ domain. In addition, those not following a GFD probably do not experience social restrictions due to the restrictive diet, which impacts their perception of the highest scores in the CDQ social domain. It is imperative to advise celiac patients who do not follow a strict GFD that they can have several health problems as a consequence of untreated CD, such as anaemia, cancer, malnutrition, osteopenia, and infertility, among others [81,82,83]. Our findings demonstrate that, in contrast to studies conducted in Poland and Morocco [8,36], where compliance with a GFD did not affect patients’ QoL, the Portuguese had a perception of QoL that was worsened in several respects. In the Polish study, the authors mentioned that it could be explained by the potential absence of gastrointestinal symptoms in non-adherent individuals [8].

Since celiac disease is chronic, the only treatment for which is the permanent withdrawal of gluten, it is essential to mobilize both the people diagnosed and their entire family and social environment so that they become a network of support and encouragement for strict maintenance of the diet. This support is a privileged component, as it promotes the emotional balance and motivational level of anyone with a chronic illness, celiac patients being no exception. Thus, adherence to and maintenance of a GFD are essential to a higher perception of QoL in all dimensions.

A potential limitation of this study was the use of a non-probabilistic convenience sample. However, if we had used random probabilistic sampling, our sample might have been smaller than what was achieved in this study. In addition, data collection during the pandemic period used the internet as the main way to reach participants. The online dissemination of the CDQ allowed for a wider distribution among the Portuguese celiac population, resulting in a larger sample without compromising the identification of the participants. Anonymous answers reduce the bias associated with the discomfort or shame of reporting GFD transgressions, allowing more accurate responses on GFD compliance and QoL perception to be obtained. The gender of the respondents can be considered another potential bias since it was not balanced (most were female). Therefore, our results do not necessarily reflect QoL perceptions for the male population in Portugal. Despite the recognized limitations on the sample representativeness obtained by the method applied in this study, this was the solution we found in order to include as many participants as possible from the entire Portuguese territory with the resources we had available.

## 5. Conclusions

Given the chronic nature of CD, there is growing interest in evaluating its outcomes and CD patients’ quality of life. Identifying the factors affecting CD patients’ perception of their QoL might assist in planning strategies to bear the burden caused by CD or its dietary treatment. The CDQ is a specific instrument that evaluates celiacs’ perceptions of their QoL, which is crucial for these patients’ care. The Portuguese CDQ demonstrated good reliability and responsiveness in this sample of Portuguese celiac patients. In Portugal, QoL perception was affected by age at diagnosis, educational level, and compliance with the gluten-free diet. Data on celiac QoL is essential to support the formulation and implementation of strategies to minimize the issues celiac patients face, thereby lowering their physical, emotional, and social burden. Moreover, data on Portuguese CD patients using CDQ will allow future comparative research to be carried out with celiac populations from different countries.

## Figures and Tables

**Table 1:** Precision of the subscales of the CDQ in Portuguese celiac patients (*n* = 234).

	Mean (SD *)	Median (IQR **)	Range	Floor Effect (%)	Ceiling Effect (%)	Internal Consistency (Alpha Cronbach)
Emotion	28.35 (7.60)	28 (23–34)	8–49	0%	1.3%	0.854
Social	23.03 (9.53)	22 (16–31)	7–49	5.1%	1.3%	0.895
Worries	26.77 (8.78)	28 (20–34)	7–49	2.6%	0.9%	0.848
Gastrointestinal	25.12 (8.81)	25 (19–33)	7–49	2.1%	1.3%	0.891
Total Score	103.28 (31.15)	103 (80–129)	29–196	0%	0.9%	0.956

* Standard deviation; ** Interquartile ranges.

**Table 2:** CDQ domains subcategorized by sex, current age, age at diagnosis, education, marital status, GFD compliance, and the use of antidepressants (*n* = 234).

	Emotion		Social		Worries		Symptoms		Total	
	Mean (SD)	*p*	Mean (SD)	*p*	Mean (SD)	*p*	Mean (SD)	*p*	Mean (SD)	*p*
Gender *										
Female (*n* = 162)	28.46 (7.11) ^a^	0.523	21.83 (9.36) ^a^	0.016	26.72 (8.65) ^a^	0.845	24.49 (8.83) ^a^	0.236	101.51 (29.65) ^a^	0.399
Male (*n* = 66)	27.68 (8.81) ^a^		25.18 (9.53) ^b^		26.47 (9.25) ^a^		26.02 (8.76) ^a^		105.35 (34.61) ^a^	
Age *										
Up to 40 y/o (*n* = 132)	28.10 (7.95) ^a^	0.565	22.66 (9.62) ^a^	0.499	26.59 (9.10) ^a^	0.718	25.02 (9.29) ^a^	0.842	102.37 (32.28) ^a^	0.614
>40 y/o (*n* = 102)	28.68 (7.15) ^a^		23.51 (9.43) ^a^		27.01 (8.38) ^a^		25.25 (8.20) ^a^		104.45 (29.75) ^a^	
Age at diagnosis *										
Up to 20 y/o (*n* = 115)	29.12 (7.86) ^a^	0.218	24.80 (9.86) ^b^	0.009	27.56 (8.68) ^a^	0.305	27.28 (8.98) ^b^	0.000	108.76 (32.62) ^b^	0.017
> 20 y/o (*n* = 116)	27.91 (7.07) ^a^		21.58 (8.82) ^a^		26.39 (8.59) ^a^		23.27 (8.09) ^a^		99.14 (28.05) ^a^	
Educational level **										
Up to elementary school (*n* = 35)	29.34 (8.92) ^a^		26.46 (9.48) ^ab^		27.17 (9.81) ^a^		28.00 (7.93) ^c^		110.97 (33.72) ^b^	
High school (*n* = 61)	30.05 (7.12) ^a^	0.104	26.49 (8.34) ^b^	0.000	28.72 (7.94) ^a^	0.186	26.74 (8.39) ^a b^	0.014	112.00 (28.43) ^b^	0.005
Undergraduate (*n* = 89)	27.51 (7.11) ^a^		21.78 (9.30) ^a^		25.97 (8.79) ^a^		24.26 (8.85) ^ab^		99.51 (30.13) ^ab^	
Post-graduation (*n* = 49)	27.06 (7.78) ^a^		18.55 (9.19) ^a^		25.53 (8.82) ^a^		22.63 (9.15) ^a^		93.78 (31.21) ^a^	
Marital status *										
With partner (*n* = 142)	28.39 (7.58) ^a^	0.927	23.28 (9.71) ^a^	0.617	27.37 (8.54) ^a^	0.195	25.31 (8.65) ^a^	0.689	104.35 (31.03) ^a^	0.513
No partner (*n* = 92)	28.29 (7.67) ^a^		22.64 (9.27) ^a^		25.85 (9.10) ^a^		24.84 (9.09) ^a^		101.62 (31.44) ^a^	
Gluten-free diet *,***										
No (*n* = 105)	28.06 (7.68) ^a^	0.596	25.18 (8.59) ^b^	0.002	27.37 (7.91) ^a^	0.340	27.45 (7.80) ^b^	0.000	108.06 (29.67) ^b^	0.034
Yes (*n* = 129)	28.59 (7.56) ^a^		21.28 (9.92) ^a^		26.29 (9.43) ^a^		23.23 (9.15) ^a^		99.39 (31.90) ^a^	
Antidepressants *										
No (*n* = 218)	28.07 (7.54) ^a^	0.036	23.03 (9.42) ^a^	0.990	26.82 (8.66) ^a^	0.783	25.18 (8.62) ^a^	0.725	103.10 (30.70) ^a^	0.743
Yes (*n* = 16)	32.19 (7.68) ^b^		23.00 (11.27) ^a^		26.19 (10.62) ^a^		24.38 (11.45) ^a^		105.75 (37.91) ^a^	

* Student’s *t*-test. ** Anova with Tukey’s posthoc. Groups with the same letters do not differ significantly. *** Compliance with a gluten-free diet was considered in participants’ responses of “always following the diet”.

## Data Availability

Data sharing is not applicable to this article.
